# Dentin-pulp complex reactions in conventional and radiation-related caries: A comparative study

**DOI:** 10.4317/jced.55370

**Published:** 2019-03-01

**Authors:** Jéssica-Montenegro Fonsêca, Natália-Rangel Palmier, Wagner-Gomes Silva, Karina-Morais Faria, Pablo-Agustin Vargas, Marcio-Ajudarte Lopes, João-Victor Salvajoli, Thais-Bianca Brandão, Ana-Carolina-Prado Ribeiro, José-Flávio-Affonso Almeida, Mario-Fernando De Goes, Alan-Roger Santos-Silva

**Affiliations:** 1 Departamento de Diagnóstico Oral, Faculdade de Odontologia de Piracicaba, Universidade Estadual de Campinas (UNICAMP), Piracicaba, São Paulo, Brasil; 2 Serviços de Odontologia e Radioterapia, Instituto do Câncer do Estado de São Paulo (ICESP), Faculdade de Medicina da Universidade de São Paulo, São Paulo, Brasil; 3 Departamento de Odontologia Restauradora, Faculdade de Odontologia de Piracicaba, Universidade Estadual de Campinas (UNICAMP), Piracicaba, São Paulo, Brasil

## Abstract

**Background:**

Radiation-related caries (RRC) is one of the most significant oral toxicities of head and neck radiotherapy (HNRT); however, the potential of radiation to directly cause harmful dentin and pulpal effects and impair response to caries progression is controversial.

**Material and Methods:**

Therefore, the aim of this study was to characterize the reactions of the dentin-pulp complex in teeth affected by RRC. Patients and methods: Twenty-two carious teeth extracted from 22 head and neck cancer (HNC) patients were divided into control (conventional caries; n=11) and irradiated (RRC; n=11) groups and paired matched by dental homology, clinical patterns of caries progression following the Post-Radiation Dental Index (PRDI) and microscopic depth of carious invasion. Histopathological characteristics based on morphological hierarchy, cell populations of dental pulp, blood vessels, neural elements, extracellular matrix components, inflammation, patterns of carious invasion and reactionary dentin presence were evaluated by optical light microscopy and histomorphometry.

**Results:**

Mean PRDI scores were 3.2 for the control group and 3.8 for the irradiated group. Dentin demineralization patterns were also similar between the groups and the mean depths of demineralization were 1,158.58µm and 1,056.89µm for the control and irradiated groups, respectively.

**Conclusions:**

Pulp histopathological changes and dentin reaction patterns were similar between groups and varied according to the PRDI scores and carious lesions depth. Dentin and pulp reactions are highly preserved in RRC teeth.

** Key words:**Cancer, radiotherapy, radiation-related caries, teeth, pulp.

## Introduction

Head and neck cancer (HNC) represent 6% of all human malignancies and approximately 650,000 new cases are annually diagnosed worldwide. Treatment protocols often involve the combination of surgery, chemotherapy, and head and neck radiotherapy (HNRT). Although considered highly effective in the loco regional control of cancer, HNRT results in a myriad of acute and chronic toxicities to non-targeted tissues, including oral mucositis, hyposalivation, recurrent oral infections, trismus, radiation-related caries (RRC) and osteoradionecrosis, among others (1,2).

RRC, also known as “radiation caries”, is a chronic side effect that affects up to 25% of patients who underwent HNRT. Its hallmark is a high potential for generalized dentition breakdown and clinical patterns of progression that differ from conventional caries, being characterized by widespread cervical demineralization, incisal edges and cusp tips lesions and diffuse brownish to black discoloration of the smooth surface of enamel. RRC rapidly progresses causing enamel cracks, delamination and amputation of teeth crowns, leading to teeth destruction. In addition, it can increase the risk for the development of osteoradionecrosis and negatively impact the overall oral function as well as the quality of life of cancer survivors (3,4). 

One of the most controversial topics in the scenario of HNRT side effects is the ability of ionizing radiation to cause direct radiogenic damage to the teeth. Although some studies have suggested that this direct radiogenic damage to structural components of the dentin and pulp, would lead to RRC (5,6), others have linked the increased risk of caries in post-HNRT patients with the indirect effects of radiotherapy (RT). These would include hyposalivation, oral microbiota alterations, impaired saliva self-cleaning properties, poor oral health status prior to and after treatment, increased dietary intake of carbohydrates, and insufficient fluoride exposure, which compose the cluster of oral symptoms that predisposes patients to rampant caries regardless of the direct effect of radiation on teeth (7,8).

In addition, to date, no in vivo study has been conducted to characterize the reactions of the dentin-pulp complex in teeth affected by RRC. 

Hence, considering that HNRT is routinely used in more than 90% of all HNC patients (2), it is paramount to precisely understand its impact on the reactions of the dentin and pulp to caries progression. Therefore, this study aimed to test the hypothesis that the *in vivo* irradiated human teeth affected by RRC have microscopically discernible effect on dentin and pulp responses when compared to conventional caries teeth samples, such as changes in the morphological pulp hierarchy, alteration in the blood vessels structure, pulp fibrosis, high incidence of calcification and necrosis and atypical pulp inflammation patterns.

## Material and Methods

-Patients and specimen collection

This study was approved by the local Ethics Committee (protocol number 023/2015) and was conducted in accordance with the Declaration of Helsinki.

Eleven irradiated teeth with RRC and eleven carious non-irradiated teeth from HNC patients were included. The sample size was determined according to the amount of extracted teeth, collected independently of the particulars of the study and that met the inclusion criteria established. Dental extractions were performed due to advanced caries or periodontal disease in both teeth groups (control and irradiated). Immediately after the extractions, teeth were identified, placed in plastic containers with 10% buffered formalin solution and fixed for at least 72 h at 4 °C (3).

For clinical characterization of the patients, the electronic medical record system was consulted and the following data were collected: age, gender, tumour topography, alcohol consumption and smoking habit, tumour histological type, clinical cancer stage 

(according the American Joint Committee on Cancer), total radiation dose prescribed to tumour treatment (Gy), anatomic origin of extracted teeth, and time between the end of HNRT and teeth extraction.

-Inclusion Criteria 

Eleven teeth affected by RRC were extracted from 11 different patients with head and neck squamous cell carcinomas (SCC) who were subjected to clinical radiation protocols with tridimensional conformal RT (3DRT) in 6-mV linear accelerators on the Synergy Platform (Elekta AB, Stockholm, Sweden) with cumulative doses that ranged from 60 to 70 Gray (Gy) (2 Gy/day, five days per week). The 3DRT plan of the patients was retrieved from the CMS system XiO version 4.60 (Elekta CMS software, St. Louis, MS, USA) to study the radiation field and the total dose directed to the teeth (9). Eleven carious non-irradiated teeth specimens were extracted from 11 different head and neck SCC patients before RT during mouth conditioning protocols.

-Exclusion Criteria

Were excluded from the study patients with SCC located in other topographies of the head and neck region, who did not receive dental treatment prior to RT, who were submitted to radiotherapy regimens different from those included in the inclusion criteria, patients being fed by nasogastric catheter, who are receiving central pain control analgesia (opioids) or whose demographic and clinicopathological information are not fully available in the medical records.

-Macroscopic analysis

All twenty-two carious teeth samples were divided into two groups: control (conventional caries; n=11) and irradiated (RRC; n=11), catalogued and subjected to photographic documentation. Teeth samples from both groups were classified and matched by dental homology (anatomic group), clinical patterns of caries progression established by the Post-Radiation Dental Index (PRDI). The PRDI index is a clinical index for assessing post-radiation dentition breakdown (10). This system is divided into five levels that range in numerical codes from 0 to 5, with the best condition represented by score 0 and the worst condition by score 5 (10).

-Demineralization and histological preparation

All specimens were cleaned up with manual periodontal curettes to remove residual soft tissues and decalcified in Ana Morse’s solution (equal volumes of 20% sodium citrate and 50% formic acid) at 4 °C for three weeks, with the solution being changed every two days. The decalcification was monitored and confirmed by weekly periapical radiographs. Specimens were sectioned along the longitudinal teeth axis through the center of the deepest carious lesions with the aid of a histologic disposable razor. The samples were embedded in Paraplast Plus® (Leica Biosystems Richmond, Inc., Richmond, IL, USA) to produce 5-µm-thick sections on a microtome (Leica, Nussloch, Germany) in silanized slides for hematoxylin and eosin (H&E) morphological evaluation.

-Optical light microscopy analysis

An optical light microscope (OLM) (DM4000 B Leica, Wetzlar, Germany) was used for the micromorphological study of the cell populations of dental pulp, blood vessels, neural elements, extracellular matrix components, inflammation, depth of carious invasion, reactionary dentin presence and patterns of demineralized dentin. Three demineralized histological sections of each specimen were analyzed and illustrative microscopic images were captured.

Two previously calibrated oral pathologists analyzed the slides. A descriptive analysis was performed for the morphologic criteria (11,12) regarding microscopic dentin and pulp reactions to caries progression, which were evaluated in a semi-quantitative way and compared between groups: presence or absence of hierarchy of the dental pulp, blood vessels and preservation of pulp extracellular matrix components through the evaluation of the presence or absence of fibrosis; high incidence of calcification, necrosis and pulp inflammation and the presence of reactionary dentin. Examiners were instructed to come to a consensus in discordant cases.

Results were analyzed by using descriptive statistics, absolute values, and percentages. Mean microscopic depth of caries invasion was quantitatively determined by measuring the distance from the surface of the demineralized dentin to the deepest point of caries affected dentin in three demineralized histological sections of each specimen, in both groups, which was obtained in microns (µm) by using the software LAS version 4.2.0 (Leica Microsystems, Switzerland).

-Statistical analysis

Morphological outcomes were descriptively analyzed and the results generated were analyzed by using descriptive statistics (Fisher’s exact test), absolute values, and percentages. Mean values of microscopic depth of caries invasion for each specimen were compared between both groups using the Student’s t-test for independent samples. The software IBM SPSS Statistics for Windows version 22.0 (Armonk, NY, USA) was used with the significance level set at α=0.05.

## Results

All patients were diagnosed with squamous cell carcinomas.

Demographic features and clinicopathological data obtained from the 22 patients are described in  Table 1. The control and irradiated groups consisted of 6 molars (54.54%), 3 pre-molars (27.27%), 1 incisor (9.09%) and 1 canine (9.09%) each. The mean time for teeth extraction following HNRT was 33 months, ranging from 4 to 62 months. The mean dose received by each tooth sample was 53.47 Gy, ranging from 38.79 to 69.33 Gy ( Table 1).

**Table 1:**
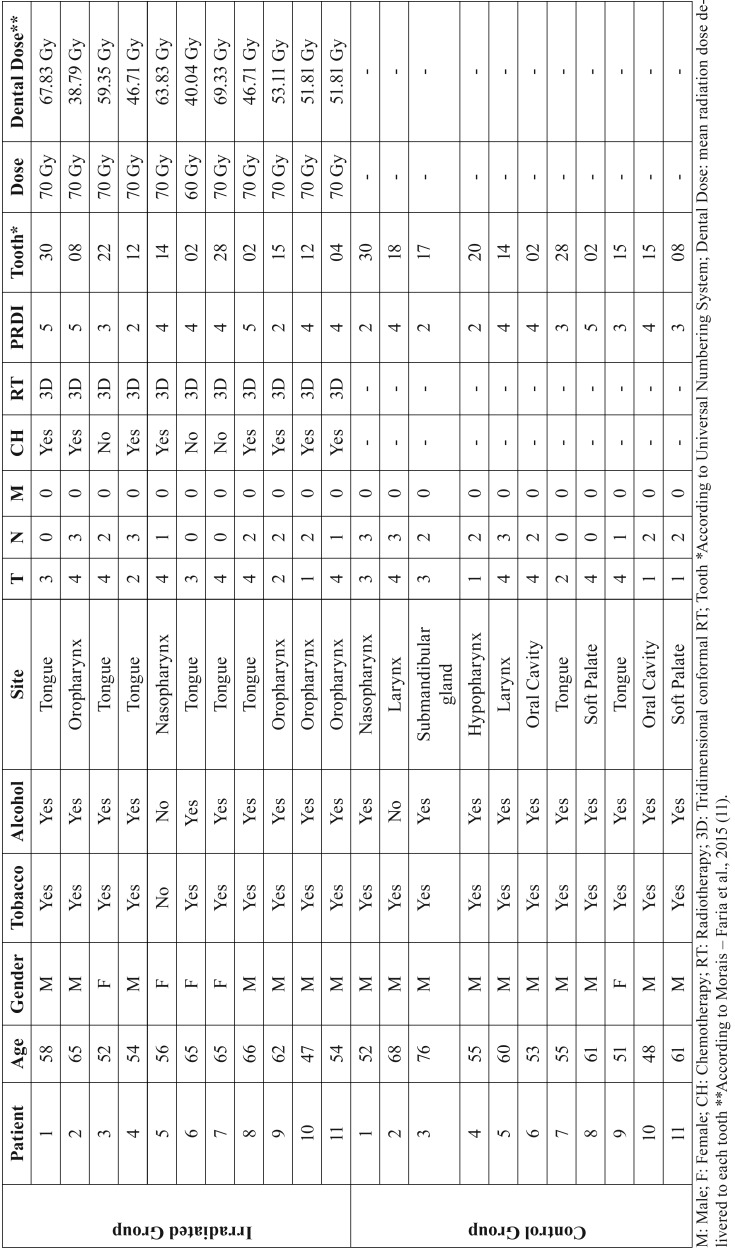
Clinicopathological profile of the patients included in the study.

Mean PRDI scores were 4 for the control group and 4 for the irradiated group, with values ranging from 2 to 5 in both groups. The dentin demineralization patterns were similar between the groups and marked by triangular demineralization with the base at the tooth surfaces and the apex pointing towards the pulp. Demineralization depth due to caries progression was controlled during specimens’ selection, leading to a mean depth of 1.158.58 μm (ranging from 471.57 to 2.498.48 μm) in the control group and 1.056.89 μm (ranging from 750.75 to 1.407.54 μm) in the irradiated group (*p*=0.79).

**Figure 1:**
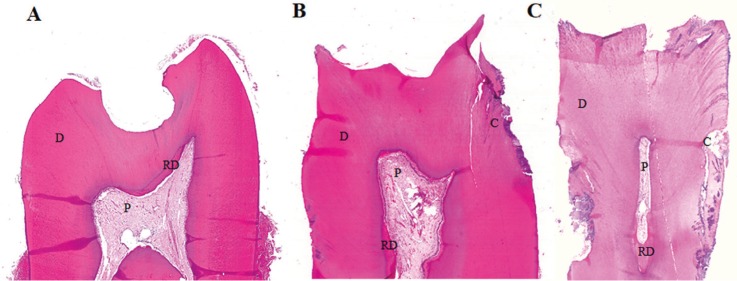
Microscopic overview of irradiated teeth crowns affected by caries showing preservation of the dental pulp (Hematoxylin and Eosin-stained sections, 2X magnification). A. Irradiated molar sample (PRDI=2) B. Irradiated premolar sample (PRDI=3) C. Irradiated premolar sample (PRDI=4). Note: Dentin (D), pulp (P), reactionary dentin (RD) and caries (C).

All pulp specimens presented a polarized odontoblastic layers arranged in palisade, subodontoblastic cell-poor layers of Weil and central zones with prominent normal blood vessels, fibroblasts, and neural bundles. But the microscopic analysis revealed the presence and the preservation of the pulp cellular layers hierarchy in 8 (72.7%) cases for the control group and 8 (72.7%) cases of the irradiated group (*p*=0.28) (Fig. 1A-C). In the other cases of both groups, the presence and the preservation of the pulp cellular layers hierarchy was changed because of calcification, diffuse chronic inflammation represented by mononuclear cells and necrosis associated to bacterial invasion. Blood vessels presence and vascular architectural preservation was observed in all (100%) samples of both groups.

**Figure 2:**
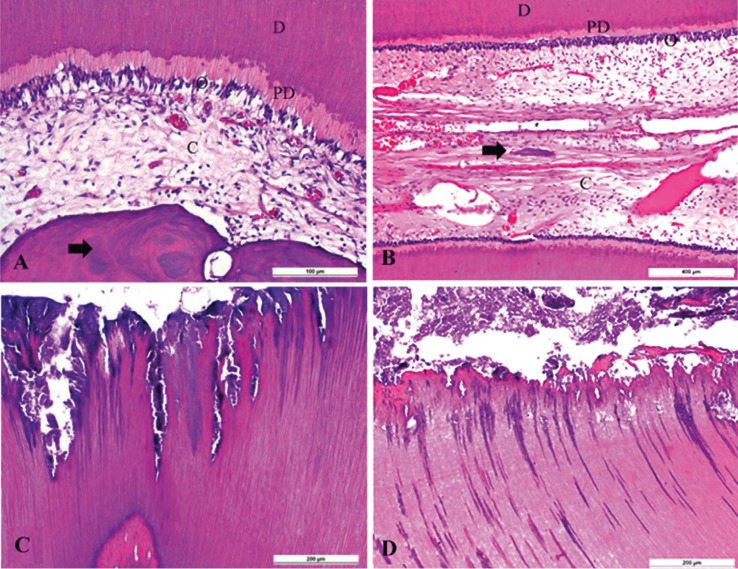
Control and irradiated samples exhibiting preservation of the dental pulp layers hierarchy and bacterial invasion (Hematoxylin and Eosin-stained sections). A. Dentin (D), predentin (PD), odontoblasts (O), pulp central region (C) and dystrophic calcification (arrow) in a control specimen B. Presence of preserved neural vascular bundles, dystrophic calcification, and hyperemia in an irradiated specimen. Dentin (D), predentin (PD), odontoblasts (O), pulp central region (C) and calcification (arrow). C. Patterns of bacterial invasion of the dentin in a control specimen. D. Caries-infected dentin composed of bacterial colonies and disorganized dentin. Inner demineralized layer with affected dentin showing normal patterns of bacterial invasion of the irradiated dentin.

Odontoblasts from all samples were characterized by tall columnar cells arranged in palisade and located at the periphery of the dental pulp. Cell processes arising from the odontoblasts cell body could be observed penetrating into the dentin and in close contact between fibroblasts of all studied samples (Fig. 2A,B).

Superficial caries-infected dentin composed of disorganized dentin and bacterial colonies, as well as an inner demineralized layer with affected, but not disrupted, dentin was consistently observed in all studied specimens of both groups (Fig. 2C,D).

**Figure 3:**
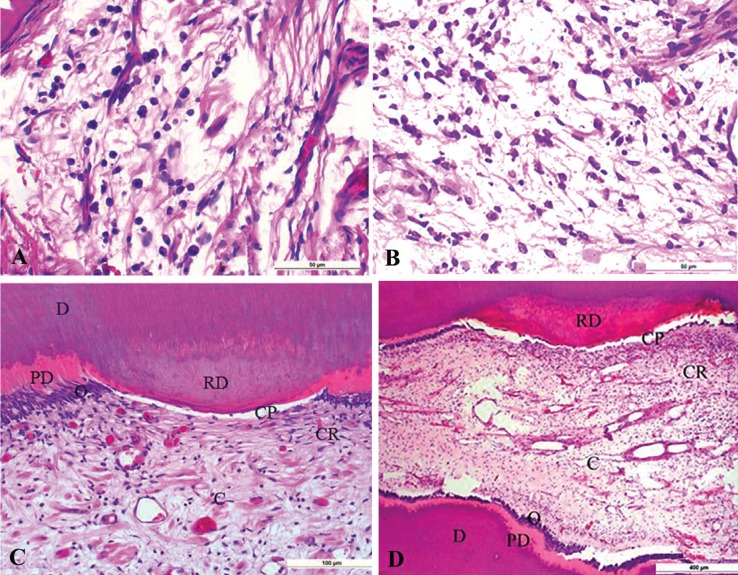
Control and irradiated samples exhibiting preservation of the dental pulp micromorphology (Hematoxylin and Eosin-stained sections). A. Chronic Inflammation represented by mononuclear cells affecting the pulp tissue of a control specimen. B. Chronic inflammation represented by mononuclear cells affecting irradiated pulp tissue. C. Preservation of the dental pulp micromorphology and dentin-pulp complex reactions to caries in a control specimen. Dentin (D), reactionary dentin (RD), predentin (PD), odontoblastic layer (O), cell-poor zone (CP), cell-rich zone (CR) and central region (C) with preserved fibroblasts, and vascular bundles. D. Preservation of the dental pulp layers hierarchy and dentin-pulp complex reactions to radiation-related caries. Reactionary dentin (RD), cell-poor zone (CP), cell-rich zone (CR), central region (C), odontoblasts (O), predentin (PD), and dentin (D). Note the presence of preserved fibroblasts and neural vascular bundles.

Pulp extracellular matrix components were similarly detected in both groups and characterized by focal areas of fibrosis: 8 (72.7%) control cases vs. 7 (63.6%) irradiated cases (*p*=0.9); calcification: 5 (45.5%) cases in each studied group (*p*=0.34); necrosis: 4 (36.4%) control cases vs. 3 (27.3%) irradiated cases (*p*=1.00); and chronic inflammation represented by mononuclear cells: 7 (63.6%) control cases vs. 6 (55.5%) irradiated cases (*p*=0.28) (Fig. 3A,B). Reactionary dentin formation was detected underlying caries demineralization fronts. The presence of reactionary dentin was observed in 6 (55.5%) cases of the control group and 5 (45.5%) cases of the irradiated group (*p*=0.28) (Fig. 3C,D).

No significant difference was encountered between irradiated and non-irradiated groups in any of the analyzed parameters. No evidence of abnormal cellular components or architecture could be detected.

## Discussion

The potential of HNRT to cause direct harmful dentin and pulpal effects that could impair the response of the dentin-pulp complex to caries progression is controversial.

There are still few studies in the literature on this point and apparently, this was the first study to compare RRC and conventional caries specimens using matched-paired teeth groups. Kataoka *et al.* (13) showed that the levels of pulp oxygenation are decreased during RT for malignant intraoral and oropharyngeal tumors. More recently, the same group of authors (14) demonstrated normal pulp oxygenation levels after 4 to 6 years of the conclusion of HNRT, suggesting that RT may not have a long-term influence on pulp vitality. Influenced by their results, we decided to perform a study to investigate microscopic evidence that RT could be directly injurious to the pulp and impair response to caries progression by using teeth extracted after a mean time of 33 months following the conclusion of HNRT (ranging from 4 to 62 months).

The clinicopathological profile of patients enrolled in this study is in accordance with the traditional features of oral and oropharyngeal SCC patients observed worldwide (15), which is marked by elderly male individuals, smokers and drinkers with poor oral health status who were diagnosed at late stages of tumor progression (16).

The clinical aggressiveness and the potential for generalized dental destruction have been well documented in RRC patients and predominately linked to post-radiation hyposalivation (3,17). However, observations based on *in vitro* studies proposed that the direct effects of HNRT on tooth-mineralized structures might also be a significant causal factor for RRC (5,7).

Although the potential benefits in terms of reducing treatment-associated toxicities, the intensity-modulated radiotherapy (IMRT) technology is not available in many centers of the world, where 3DRT is still routinely used (18). In this context, 3DRT was the technology used in all of the patients investigated in the present study and its use is still widely accepted because no overall survival benefits have been observed in HNC patients treated with IMRT (19). Most importantly, high doses of radiation were directed to the irradiated group samples of the current study (mean dose of 53.47Gy) and the influence of the radiation technique should not be considered an issue in terms of radiogenic effects, especially because a collaborative study recently demonstrated that IMRT and 3DRT deliver similar doses of radiation to the teeth of HNC patients (20).

One of the limitations of the current study was the small number of teeth specimens analyzed; wherein larger sample sizes would probably lead to more robust results. In addition, another limitation was that there were different teeth assessed in the irradiated and control groups due to the impossibility of analysing *in vivo* irradiated teeth in different periods of time. However, it is important to clarify that this limitation was minimized by using a strict methodological design, including a paired-matched sampling approach by teeth anatomic origin, clinical stage of RRC progression (through the PRDI) and microscopic depths of caries invasion. In addition, this study relied on human *in vivo* irradiated teeth (which are seldom collected because of the risk of osteoradionecrosis) rather than in samples irradiated *in vitro*. Comparisons between studies that evaluated *in vivo* and *in vitro* effects of RT on dental pulp should be carefully established because *in vitro* simulated RT does not represent real clinical conditions concerning the cariogenic microenvironment or the dosimetric standards for HNRT (8).

The PRDI (10) was created based on the pilot macroscopic study supported by ex vivo digital photographs of the dental surfaces affected by RRC lesions, where a group of specialists evaluated criteria for caries diagnosis and dental destruction. Although the PRDI isn’t the gold standard for the diagnosis of RRC or conventional caries, its features regarding the amount of structure loss of the samples was a valuable tool to pair matches the samples regarding the clinical patterns of caries progression.

From a microscopic point of view, patterns of dentin demineralization presented normal architecture (9,10) and varied according to the PRDI in both groups. The microscopic analysis found homogeneous results between the groups, showing no statistical difference to the criteria presence or absence of hierarchy of the dental pulp, blood vessels and preservation of pulp extracellular matrix components through the evaluation of the presence or absence of fibrosis; high incidence of calcification, necrosis and pulp inflammation and the presence of reactionary dentin.

Previous studies based on animal experimental models or with an obsolete technique of RT, like radiation by a cobalt-60-source, also investigated pulpal reactions related to radiation, but not to caries progression, and concluded that only teeth subjected to high doses of radiation (more than 50 Gy) showed alterations in the dental pulp tissue, including fibrotic and inflammatory degenerations (21,22). These authors also correlate alterations such as damage of tooth hard tissue, degradations of the organic substances in the enamel, destruction of vital cells in the dentin and increased acid solubility, as a direct effect of the RT (1,4).

On the other hand, previous studies defend the theory that the indirect effects of HNRT on dental structure, such as dryness caused by the hyposalivation due to damage to the salivary glands, the fracture toughness of enamel decrease and the enamel becomes more brittle. Dehydration also affects the dentin, so the teeth are more susceptible to cracking (23,24). In supplement to salivary changes, post-HNRT patients also have a more cariogenic diet, which leads to a low oral pH and consequently to dental demineralization, consolidating the idea that the “clustering of oral changes” caused by treatment, especially the reduction of salivary flow, might be responsible for an onset and progression of RRC (8,23-25).

In conclusion, the present study rejected the hypothesis that HNRT is able to impair the micromorphological pulp reactions to RRC progression. Therefore, direct effects of radiation may not be regarded as an independent factor to explain the rapid onset and aggressive clinical patterns of RRC progression.
